# A single-center observational study of nirsevimab for prevention of RSV infection in preterm infants

**DOI:** 10.3389/fped.2026.1841732

**Published:** 2026-07-08

**Authors:** Rina Su, Rigonggaowa A, Fang Yao, Yanbin An, Cheng Cai, Lei Yun

**Affiliations:** 1 Department of Neonatology, Inner Mongolia Maternity and Child Health Gare Hospital, Hohhot, Mongolia; 2 Department of Pharmacy, Inner Mongolia Maternity and Child Health Gare Hospital, Hohhot, Mongolia; 3 Department of Neonatology, Shanghai Children’s Hospital, School of Medicine, Shanghai Jiao Tong University, Shanghai, China

**Keywords:** effectiveness, nirsevimab, preterm infants, respiratory syncytial virus infection, safety

## Abstract

**Objective:**

This study aims to evaluate the clinical efficacy of nirsevimab in preventing respiratory syncytial virus (RSV) infection in preterm infants, highlighting its potential to improve neonatal respiratory health.

**Methods:**

We conducted a retrospective cohort study involving preterm infants admitted to the Neonatology Department of Inner Mongolia Maternity and Child Health Care Hospital between November 2024 and January 2026. The immunization group included infants receiving nirsevimab, while the non-immunized group was matched via propensity score matching. Both groups received standard care. Clinical data, as well as diagnostic and therapeutic processes, were collected. The efficacy and safety of nirsevimab were evaluated, and the incidence and severity of RSV-associated respiratory infections in two groups were followed up (severe cases were defined as those requiring respiratory support or experiencing adverse outcomes).

**Results:**

(1) Compared with the non-immunized group, the efficacy rate of preventing RSV-associated respiratory infections was significantly higher in the nirsevimab immunized group (98.73% vs. 84.81%), with a statistically significant difference (*p* < 0.05). (2) In the non-immunized group, twelve preterm infants (15.18%) developed RSV-associated respiratory infections, among whom 4 cases (5.06%) required non-invasive ventilator support, 2 cases (2.53%) experienced myocardial injury, 1 case (1.26%) had liver injury, 1 case (1.26%) experienced myocardial injury combined with liver injury, and 1 case (1.26%) exhibited wheezing symptoms. (3) All preterm infants infected with RSV in the non-immunized group required rehospitalization, with an average hospital stay of 15.5 days; in contrast, no rehospitalization was required in the immunized group. (4) No significant adverse reactions were observed in the immunized group following nirsevimab injection.

**Conclusions:**

During the RSV epidemic season, our findings indicate that prophylactic treatment with nirsevimab significantly reduces the incidence of RSV infection in preterm infants, decreases the rehospitalization due to RSV infection in preterm infants, and demonstrates a favorable safety profile.

## 1 Introduction

Respiratory syncytial virus (RSV) is one of the primary pathogens responsible for respiratory tract infections in newborns, particularly acute lower respiratory tract infections (1). Following RSV infection, newborns may exhibit symptoms such as tachypnea and wheezing; in severe cases, these symptoms can progress to asthma. In preterm infants, apnea is often the predominant clinical manifestation of RSV infection (2), and in severe instances, respiratory failure may occur, potentially necessitating mechanical ventilation (3). The “Chinese Expert Recommendations on the Use of Nirsevimab in Infants” (4) state: “Currently, there is no drug available for the prevention of RSV in children, making non-pharmaceutical interventions an effective means of preventing RSV infection in newborns”. To enhance the prevention of RSV infection, the World Health Organization has integrated RSV drug development into its global drug strategy and initiated extensive clinical research (5). Internationally, in the late 1990s, palivizumab received its first approvals for the prevention of severe respiratory syncytial virus (RSV) disease in high-risk children (6). However, this antibody has not been introduced in China. Fortunately, following the approval of palivizumab, nirsevimab has received approval in multiple countries and, as of January 2024, has become the first and only passively immunizing antibody approved in China for the prevention of RSV infection in infants.

Nirsevimab is a monoclonal antibody with a prolonged elimination half-life that specifically targets the respiratory syncytial virus (RSV), demonstrating efficacy that is approximately 50 times greater than that of palivizumab. It is primarily used to prevent RSV-related lower respiratory tract infections in newborns and infants. By targeting the prefusion structure of the RSV fusion (F) protein, nirsevimab blocks viral entry into host cells, providing passive immunisation (7). This medication is recommended for newborns and infants with pre-existing health issues (8). According to the “Expert Recommendations on the Use of Nirsevimab in Infants in China” (4): monoclonal antibodies can be employed to prevent RSV infections in infants under one year old during the RSV season. Most research regarding nirsevimab's effectiveness in preventing RSV in infants has been conducted in Europe and the United States, with a notable absence of similar studies in China. Consequently, assessing nirsevimab's effectiveness in Chinese infants is crucial. Infants who are low birth weight, born prematurely, or have congenital heart conditions are particularly vulnerable to respiratory infections. Preterm infants are especially at risk for RSV due to their narrower airways, immature immune systems, lower levels of maternal antibodies, and a higher likelihood of chronic lung conditions like bronchopulmonary dysplasia (9). Additionally, RSV infections in these infants can result in more severe health issues. This research evaluates the clinical effectiveness of nirsevimab in preventing RSV infections in preterm infants, aiming to establish a clinical foundation for RSV prevention in this at-risk population.

## 2 Material and methods

### 2.1 General information

Between November 2024 and January 2026, we selected 158 preterm infants from the Neonatology Department of Inner Mongolia Maternity and Child Health Gare Hospital for this study. Preterm infants receiving nirsevimab immunization were defined as the immunization group, and the non-immunization group was selected via propensity score matching (PSM).

The criteria for inclusion included: 1) Preterm infants with a gestational age less than 37 weeks (including those with a gestational age less than 32 weeks). 2) Preterm infants either entering or having been born in their initial RSV season. 3) Preterm infants who were administered nirsevimab during the RSV season.

The criteria for exclusion included: 1) Previous allergic reactions to nirsevimab. 2) Preterm infants who have thrombocytopenia, bleeding disorders, or are undergoing anticoagulant treatment. 3) Preterm infants with significant developmental abnormalities or metabolic synthesis disorders.

The Ethics Committee of the Maternal and Child Health Care Hospital in the Inner Mongolia Autonomous Region approved this research. Approval No. [2025] Ethical Review K [051-1].

### 2.2 Methods

Two groups of preterm infants were provided with symptomatic care, which included respiratory and nutritional support tailored to their specific clinical presentations. Their health conditions were continuously observed, and treatment strategies were modified as needed. Furthermore, the immunized group of preterm infants was administered nirsevimab (produced by Patheon Manufacturing Services LLC; reference: JS20230047) during the RSV season. The dosing regimen was as follows:

A single dose of 50 mg for patients weighing less than 5 kg, and 100 mg for those weighing 5 kg or more (4).

### 2.3 Observation indicators

(1) Effectiveness measures: ① Assessment of the rate of respiratory infections linked to RSV in two groups. ② Evaluation of the severity of RSV-related respiratory infections and the occurrence of complications in two groups. ③ Analysis of the length of rehospitalization caused by RSV-related respiratory infections across the two groups.
(2) Safety metrics: Monitoring for adverse events in the immunized cohort after the administration of nirsevimab, including symptoms like rashes, fever, and localized reactions such as redness, swelling, warmth, or discomfort at the site of injection.

### 2.4 Statistical processing

Statistical analysis was conducted with SPSS version 23.0, Fisher's exact test was applied due to the small sample size and low event rate. Continuous data are reported as (*x* *±* *s*), and comparisons between groups were carried out using the independent samples *t*-test. A *p*-value of less than 0.05 was deemed statistically significant.

## 3 Results

### 3.1 Analysis of general characteristics of Two groups of preterm infants

The research involved two groups of preterm infants, each containing 79 participants. The immunized group had 44 boys and 35 girls, with an average gestational age of (29 ± 1.5) weeks and birth weights between 580 and 2,510 g, averaging (1,384 ± 423.85) grams. In contrast, the non-immunized group included 43 boys and 36 girls, with a mean gestational age of (31.5 ± 3.5) weeks and birth weights ranging from 990 to 2,750 g, averaging (1,653.6 ± 716) grams.

Two groups showed similar characteristics regarding gestational age, gender, and birth weight, with no significant statistical differences observed (*P* > 0.05). (See Table 1).

**Table 1:** Comparison of general characteristics between the immunized and Non-immunized groups of preterm infants.

Category		Immunized group (*n* = 79)	Non-immunized group (*n* = 79)	*p*-value
Gestational				
Age (weeks)		29 ± 1.5	31.5 ± 3.5	0.746
Gender	Male	44	43	
	Female	35	36	0.385
Birth weight (g)		1,384 ± 423.85	1,653.6 ± 716	0.499

### 3.2 Comparison of the occurrence rate of RSV-associated respiratory tract infection between the two groups

The RSV infection rate was significantly lower in the nirsevimab group compared with the non-immunized group group (1.27% vs. 15.19%). The difference between groups was statistically significant by Fisher's exact test (*p* = 0.001). The efficacy rate was 98.73% in the nirsevimab group vs. 84.81% in the non-immunized group group. (See Table 2).

**Table 2:** Comparison of RSV-associated respiratory infections between the two groups of preterm infants [*n* (%)].

Group	*N*	Clinical outcome			
		RSV (+)	RSV (−)	Effectiveness rate (%) (95% CI/CrI)	*p*-value
Immunized	79	1	78	0.07 (0.01–0.57)	0.0012
Unimmunized	79	12	67		

### 3.3 Comparison of the severity of RSV-associated respiratory tract infections between the two groups

Within the immunized group, out of 79 preterm infants, 78 cases did not experience any respiratory tract infections linked to RSV throughout the follow-up duration, only one preterm infant developed RSV infection during the first RSV season after discharge and required hospitalization.

Within the non-immunized group, twelve preterm infants (15.18%) developed RSV-associated respiratory infections, among whom 4 cases (5.06%) required non-invasive ventilator support, 2 cases (2.53%) experienced myocardial injury, 1 case (1.26%) had liver injury, 1 case (1.26%) experienced myocardial injury combined with liver injury, and 1 case (1.26%) exhibited wheezing symptoms.

In contrast to the immunized group, every preterm infant in the non- immunized group who contracted an infection needed to be readmitted to the hospital, averaging a stay of 15.5 days, but all were successfully treated and released. Conversely, within the immunized group, none of the 78 infants experienced RSV-related respiratory infections or required rehospitalization, only one preterm infant developed RSV infection during the first RSV season after discharge and required hospitalization.

### 3.4 Safety observation of nirsevimab administration in the immunized group

In the group of 26 preterm infants treated with nirsevimab for prophylaxis, no side effects were observed, including rash, fever, or any signs of redness, swelling, warmth, or discomfort at the injection site.

## 4 Discussion

Infection with respiratory syncytial virus (RSV) represents a significant public health challenge worldwide. The virus's prevalence among infants and the complications it causes place a heavy strain on families and healthcare systems alike. In northern China, RSV predominantly spreads during the winter and spring months, specifically from October to March of the following year, with a peak between November and December. There are notable differences in RSV patterns between northern and southern regions in China. This virus is the primary cause of acute lower respiratory tract infections in infants under one year old. It is a leading reason for hospital admissions in children under two years old, significantly impacting healthcare resources across our country. Research from Australia indicates that the highest rates of hospitalization for RSV-related respiratory illnesses occur in infants aged 0–3 months, with an incidence of 24.7–31.7/1,000 (10). Newborns have specific respiratory system characteristics: (1) narrow airways that increase resistance; (2) a rich blood supply in the respiratory mucosa combined with limited ciliary movement; (3) soft, pliable airway cartilage. These traits lead to a higher frequency of respiratory infections in infants, often resulting in respiratory distress. Newborns those with low birth weight, and those with congenital heart conditions are particularly vulnerable to respiratory infections (11). Consequently, preterm infants infected with RSV face a greater risk of developing severe pneumonia and complications like acute asthma.

Currently, there is no dedicated treatment for RSV infections in newborns, underscoring the importance of preventive measures. Despite extensive global initiatives and research aimed at developing an RSV drug over the years, a safe and effective option remains unavailable. Consequently, passive immunization is a vital approach to safeguarding infants from RSV infections. The “Chinese Expert Recommendations on the Use of Nirsevimab in Infants” (4) endorse nirsevimab for preventing RSV in newborns. Administering it during the initial RSV season can significantly reduce the risk of lower respiratory tract infections associated with the virus. RSV is an enveloped RNA virus categorized into two subtypes, A and B, based on their antigenic properties. RSV virus comprises 11 proteins derived from 10 genes, with the fusion (F) and attachment (G) proteins being the primary targets for neutralizing antibodies (12). The mechanism of action of nirsevimab is to block RSV entry into cells. It binds to a conserved epitope on the F protein, specifically engaging the F1 and F2 subunits of the fusion complex, thereby stabilizing the F protein in its pre-fusion state and hindering the virus's ability to infect host cells (7). Griffin et al. in 2020 (13) revealed that administering nirsevimab prior to the RSV season for high-risk infants (gestational age 29–35 weeks) resulted in a 70.1% efficacy rate in preventing RSV infections.

### 4.1 Nirsevimab is highly effective in preventing RSV infections among preterm infants

This study's findings reveal that the immunized group had a markedly greater success rate in avoiding RSV-related respiratory infections compared to those who were not immunized (98.73% vs. 84.81%). Out of 79 preterm infants who received the drug, 78 cases remained free from RSV-related respiratory infections during the peak RSV season, resulting in an efficacy of 98.73%. Additionally, nirsevimab did not lead to an increase in lower respiratory infections caused by non-RSV pathogens, only one preterm infant developed RSV infection during the first RSV season after discharge and required hospitalization. These results suggest that nirsevimab effectively protects preterm infants from RSV without raising the risk of infections from other respiratory agents. Nirsevimab is a fully human, recombinant monoclonal antibody that achieves its maximum serum concentration within a week of administration, has a half-life of 63–73 days, and sustains elevated serum levels for about five months, ensuring prolonged protection against RSV in newborns (7). Phase III clinical trials indicated a 71% decrease in RSV-related respiratory infections after nirsevimab treatment, with hospitalization rates dropping by 78% in preterm infants and 75% in full-term infants (14). Therefore, nirsevimab proves to be an effective measure against RSV infections in preterm infants.

Preterm infants who did not receive nirsevimab were at risk for respiratory tract infections linked to RSV. In the study's unimmunized cohort of 79 preterm infants, twelve preterm infants (15.18%) developed RSV-associated respiratory infections, among whom 4 cases (5.06%) required non-invasive ventilator support, 2 cases (2.53%) experienced myocardial injury, 1 case (1.26%) had liver injury,1 case (1.26%) experienced myocardial injury combined with liver injury, and 1 case (1.26%) exhibited wheezing symptoms. All infected preterm infants in this group required readmission to the hospital, averaging a stay of 15.5 days. The mechanisms behind RSV infection are intricate, involving various viral elements, transcriptomic and proteomic factors, influences from airway epithelial cells, immune and neurological responses, as well as host and environmental factors (15). RSV primarily targets the respiratory system, causing airway obstruction, bronchial smooth muscle contractions, and increased airway sensitivity. Additionally, RSV infection can lead to complications that extend beyond the lungs. Research into viral myocarditis in children has identified N-terminal pro-brain natriuretic peptide (NT-proBNP) as a marker for the severity of RSV-related bronchiolitis and the onset of acute heart failure (16). Another investigation noted a temporary rise in aspartate aminotransferase levels (17), occurring in 46%–49% of infants on mechanical ventilation. Ivey KS et al. (18) found RSV in postmortem analyses of cerebrospinal fluid, heart tissue, peritoneal fluid, liver, and brain in cases of fatal RSV infections. In this research, it was observed that preterm infants who did not receive vaccinations were vulnerable to respiratory infections linked to RSV and also experienced damage to organs unrelated to respiration after the infection, aligning with the previously mentioned results.

### 4.2 Nirsevimab exhibited an excellent safety profile, with no notable adverse effects reported

In this study involving 79 preterm infants treated with nirsevimab, there were no occurrences of adverse reactions such as rash, fever, injection site redness, swelling, heat, or pain, nor were there any related complications like thrombocytopenia. The rate of adverse reactions associated with nirsevimab was minimal. Possible side effects may include rash, fever, and localized reactions at the injection site, such as redness, swelling, heat, and pain, typically appearing approximately one week after injection, along with rare instances of hypersensitivity and thrombocytopenia (19). In 2025 Wu PP et al. (12) reviewed eight safety studies across different countries regarding nirsevimab's use for RSV prevention in infants and found no serious adverse events, further supporting nirsevimab's positive safety profile.

### 4.3 Constraints of this research

This investigation employed a modest sample size and was conducted at a single location. Moreover, the study's timeframe was brief, focusing solely on the immediate impact of the vaccination initiative. There is also a lack of comprehensive data concerning vaccinations in later seasons of RSV. Future research should seek to increase the sample size, prolong the follow-up duration, and adopt a multicenter approach.

To conclude, the preventive use of nirsevimab throughout the RSV outbreak period significantly lowers the occurrence of respiratory infections linked to RSV in preterm infants, minimizes the chances of being readmitted to the hospital due to RSV, and eases the considerable strain on both families and the community. Given its positive safety record and absence of major side effects.

## Data Availability

The original contributions presented in the study are included in the article/Supplementary Material, further inquiries can be directed to the corresponding authors.
